# PSYCHOACOUSTICS-WEB: A free online tool for the estimation of auditory thresholds

**DOI:** 10.3758/s13428-024-02430-3

**Published:** 2024-05-06

**Authors:** Massimo Grassi, Andrea Felline, Niccolò Orlandi, Mattia Toffanin, Gnana Prakash Goli, Hurcan Andrei Senyuva, Mauro Migliardi, Giulio Contemori

**Affiliations:** 1 Department of Information Engineering, University of Padua, Padua, Italy; 2 Deparment of General Psychology, University of Padua, Via Venezia 8, 35131 Padua, Italy

**Keywords:** Psychoacoustics, Hearing, Auditory threshold, Psychophysics

## Abstract

PSYCHOACOUSTICS-WEB is an online tool written in JavaScript and PHP that enables the estimation of auditory sensory thresholds via adaptive threshold tracking. The toolbox implements the transformed up-down methods proposed by Levitt (*Journal of the Acoustical Society of America*, *49*, 467-477, (1971) for a set of classic psychoacoustical tasks: frequency, intensity, and duration discrimination of pure tones; duration discrimination and gap detection of noise; and amplitude modulation detection with noise carriers. The toolbox can be used through a common web browser; it works with both fixed and mobile devices, and requires no programming skills. PSYCHOACOUSTICS-WEB is suitable for laboratory, classroom, and online testing and is designed for two main types of users: an occasional user and, above all, an experimenter using the toolbox for their own research. This latter user can create a personal account, customise existing experiments, and share them in the form of direct links to further users (e.g., the participants of a hypothetical experiment). Finally, because data storage is centralised, the toolbox offers the potential for creating a database of auditory skills.

## Introduction

In this paper, we introduce PSYCHOACOUSTICS-WEB, a new online tool written in JavaScript and PHP that enables auditory threshold estimation through adaptive threshold tracking. The tool implements adaptive procedures from the staircase family, specifically the “transformed up-down” methods proposed by Levitt (1971). Adaptive threshold tracking is available for a set of default, classic experiments measuring sensitivity in basic auditory abilities such as frequency, intensity, and duration discrimination of pure tones, duration discrimination of noise, gap detection in noise, and amplitude modulation detection with a noise carrier. PSYCHOACOUSTICS-WEB is freely accessible and only requires a web browser for operation. The toolbox is suitable for both traditional laboratory testing and remote testing, and it is compatible with personal computers as well as mobile devices like tablets or smartphones.

## On sensory sensitivity and sensory threshold estimation

Here we provide a brief theoretical overview on sensory sensitivity and sensory threshold estimation, in particular focusing on those concepts that are necessary to understand the toolbox and the way it works. The reader familiar with these concepts may wish to skip this section. Alternatively, for the reader that would like to deepen their understanding of these same concepts, we suggest volume 63 (issue 8) of *Perception & Psychophysics* (year 2001), which is completely dedicated to sensory threshold estimation. More recent theoretical updates can be found in García-Pérez (1998, 2002, 2009) or Ulrich and Vorberg (2009).

Sensation moves within and across two types of thresholds: detection and discrimination. The detection threshold is the minimum detectable stimulus level in the absence of any other stimuli of the same sort. The discrimination threshold is the minimum detectable difference between two stimuli levels. The threshold is not a fixed division between audible and inaudible. Rather, it is a gradual change in the sensation elicited by a stimulus (or in the comparative sensations elicited by two stimuli). It is for this reason that the threshold is described in probabilistic terms. For example, the threshold might correspond to the stimulus intensity that is detected 50% of the time, or the difference in intensity that is correctly identified 70.7% of the time. The value of this performance changes according to various factors, such as the type of task or the type of method used to estimate the threshold (see below). In brief, the threshold is an arbitrary point p included between floor and ceiling sensory performance, and when we estimate a threshold, we search for the stimulus level eliciting that performance p.

Thresholds can be estimated either via yes/no tasks or via multiple-alternative forced-choice tasks (in brief, *n*AFC, with *n* being the number of alternatives). The yes/no task collects self-report responses: the participant is presented with one stimulus and asked to report whether they have detected the stimulus (yes) or not (no). In contrast, *n*AFC tasks gather correct and incorrect responses: the participant is presented with one or more stimuli and is asked to report whether the stimulus (or which stimulus) has a certain characteristic among a number *n* of response alternatives. For example, in a vision experiment, we could ask the observer whether a certain stimulus is tilted to the left or to the right (i.e., 2AFC task). In audition, because stimuli are often delivered in temporal succession, the alternatives may be presented over multiple intervals, such as when we ask a listener to indicate the higher-pitch tone in a sequence of two tones. In practice, auditory research uses multiple-interval, multiple-alternative tasks (i.e., in brief, mI-*n*AFC). Note that the number of intervals and the number of response alternatives are independent, such as when we present a fixed reference tone followed by a pair of tones and ask which tone of the pair is identical to the fixed reference tone (i.e., 3I-2AFC task).

Thresholds can be estimated by means of two classes of procedures: adaptive and non-adaptive. In non-adaptive procedures, the stimuli are pre-set before the beginning of the experiment. This type of procedure will not be further discussed here. In adaptive procedures, in contrast, the stimuli levels are selected at the same time as the experiment is running, as a function of the participant's responses and according to a specific algorithm. Adaptive procedures can be grossly divided into two types: nonparametric and parametric. The major difference between nonparametric and parametric adaptive procedures is in the number of assumptions supporting the algorithm, and as the name suggests, nonparametric procedures make fewer assumptions than the parametric ones. Examples of nonparametric adaptive procedures are the method of limits by Fechner (1889), the simple up-down by von Békésy (1947), and the transformed up-down by Levitt (1971). Examples of parametric procedures are the PEST (Parameter Estimation by Sequential Testing) by Taylor and Creelman (1967), “best” PEST by Pentland (1980), QUEST by Watson and Pelli (1983), and maximum likelihood (Green 1990, 1993). Nonparametric procedures are generally more commonly used than parametric ones, even if they have some disadvantages (e.g., Amitay et al., 2006; Leek, 2001). The reason for this preference is that nonparametric procedures are theoretically simpler and require less calculation, whilst parametric procedures are theoretically more complex and may require substantial calculation. PSYCHOACOUSTICS-WEB implements the transformed up-down methods proposed by Levitt (1971). This family of nonparametric adaptive procedures is perhaps the most widely used in psychophysics: the paper by Levitt has about 4000 citations in Scopus at the time of writing.

### The transformed up-down methods (Levitt, 1971)

The transformed up-down methods can be used to estimate both detection and discrimination thresholds. Because these methods have been used for decades, they have been investigated extensively (e.g., García-Pérez, 1998, 2002, 2009; Treutwein, 1995) and compared with more contemporary adaptive procedures (for example, in auditory research, Amitay et al., 2006; Kollmeier et al., 1988; Marvit et al., 2003). These investigations revealed that the transformed up-down methods are a very reliable way to estimate sensory thresholds. In some cases, the authors suggested possible improvements (e.g., Brown, 1996; García-Pérez, 2009). However, these improvements had little impact in everyday laboratory use. For this reason, here, transformed up-down methods are described in their most commonly used variants. They are also implemented in their most common variants in PSYCHOACOUSTICS-WEB.

Let us consider the case in which we wish to estimate the frequency discrimination threshold of a 1-kHz pure tone. We will present a series of trials, and in each trial there will be two stimuli: the standard and the variable. The frequency of the standard is fixed. The frequency of the variable is higher than the standard of a certain value Δf. The value of Δf will be adaptively changed during the experiment as a function of the participant’s response. In each trial, the standard and variable are presented in random order and the listener is tasked with identifying the tone with the higher pitch (i.e., a 2I-2AFC task). Levitt (1971) proposes grouping responses in “up” and “down” categories. For example, if we follow the two-down/one-up rule, Levitt (1971) suggests reducing the value of Δf after two consecutive correct responses (i.e., after a “down” group) whilst increasing the value of Δf after one incorrect response, or after one positive response followed by a negative response (i.e., after an “up” group). “Up” and “down” refer to the change in Δf when we represent it as a function of the trial number. The “down” motion of the adaptive procedure reduces the value of Δf during the trials. In contrast, the “up” motion increases the value of Δf over the trials. Levitt (1971) proposed several methods. However, the two most commonly used are the two-down/one-up and the three-down/one-up. In the three-down/one-up, the threshold tracking moves down after three consecutive positive responses and up after one negative response, or after one positive response followed by one negative response, or after two positive responses followed by one negative response. The different rules track different performance levels—or, in classic psychophysics terminology, different thresholds. Let us suppose that we are using a two-down/one-up rule and that the probability of a stimulus giving rise to a positive response is *p*. In this case, the transformed up-down method suggests moving down when the participant returns two or more positive responses and moving up when the participant produces one negative response or one positive response followed by one negative response. Therefore, the probability of moving down is *p*^2 whereas the probability of moving up is either 1 −*p* (i.e., one negative response only) or *p*(1 −*p*), i.e., one positive response followed by one negative response. In synthesis:

$${{\text{p}}}^{2}=\left(1-{\text{p}}\right)+\left(1-{\text{p}}\right)={{\text{p}}}^{2}=0.707$$

It is for this reason that the “two-down/one-up” rule tracks 70.7% of the participant’s performance. Mutatis mutandis, the three-down/one-up tracks 79.4% of the participant’s performance, and a hypothetical four-down/one-up tracks 84.1% of the participant’s performance. Transformed up-down methods become particularly interesting when the algorithm is used in a forced-choice task. In forced-choice tasks, floor performance is equal to chance level, and chance level is the reciprocal of the number of alternatives. For example, in a 2AFC task, floor performance is 50%, in a 3AFC task floor performance is 33%, and so on. In *n*AFC tasks, when the stimulus level is extremely low, the participant can still guess the correct response by chance. However, because transformed up-down methods track higher performance (i.e., 70.7% or 79.4%), the researcher can collect a sensory measure that does not mix the sensory measurement with the measurement error due to the task (see Taylor, 1971; Green, 1990; Treutwein, 1995; Leek, 2001; Kollmeier et al., 1988).

Let us assume that we began tracking the threshold with a fairly large Δf, and therefore with relatively easy trials, a fairly common practice in audition. Let us also assume we are using a two-down/one-up rule. If we apply the rule, we will observe several “downs”, several reductions of Δf. Eventually, the listeners will produce an “up” group of responses, which is one negative response or one positive response followed by a negative response. This occurrence is termed a "reversal". In the context of the transformed up-down methods, a “reversal” denotes a change in the direction of the adaptive track, a change from a “down” group of responses to an “up” group of responses (or vice versa). Note that "reversal" is not simply a change in the outcome of one (or more) trials in a sequence of trials: for instance, if we are working under the two-down/one-up/one-up rule, three positive responses (+) within a sequence like “ + − + − + −” do not constitute a reversal. In classic psychophysics, the reversal coincides with the sensory threshold of the participant, because we switch from a down group of responses to an up group of responses (or vice versa). In practice, the reversal point is, hypothetically, the value of the physical stimulus that is thought to elicit a number of positive responses that is midway between ceiling and floor: the threshold.

If we use the adaptive methods proposed by Levitt (1971), there is one main way by which we can change the stimulus level during the threshold tracking: by multiplication/division. We define “factor” as the factor by which we multiply (or divide) the value of the current delta when we need to change its value. For example, let us suppose we are estimating the frequency discrimination threshold for a 1-kHz pure tone. We may set the frequency of the standard tone to 1 kHz and the frequency of the variable tone to 1 kHz plus a certain Δf (e.g., 100 Hz). Let us suppose we set the factor to “2”. If we are approaching the threshold from above, every time we observe a down group of responses, we have to divide Δf by 2. On the contrary, if we observe an up group of responses, we have to multiply Δf by 2. In laboratory practice it may be convenient to use more than one factor in threshold tracking. In psychoacoustics, it is common to use two factors: a large one (e.g., 2), to approach the threshold quickly and a smaller one (e.g., square root of 2), for fine threshold estimation. Another common practice is to adopt a large step size in the first four reversals and a small one in the last eight (or 12) reversals. In any case, factors should not be chosen to be excessively large or excessively small: one that is too large would produce an alternation of very easy trials and very difficult trials; one that is too small would increase the sensitivity of the threshold estimation but at the cost of lengthening the experiment.

How is the threshold calculated? The transformed up-down algorithm drives the selection of the stimulus we have to deliver to the participant in a given trial. However, eventually, the experimenter may be interested in calculating a single value that represents the participant's performance. There are several options at this stage. A classic approach is to divide the threshold tracking into so-called runs. A run consists of a sequence of changes in stimulus level in one direction only. Levitt (1971) suggests calculating the midpoints of runs (i.e., the arithmetic mean of the two reversals marking the beginning and end of a run), then calculating the arithmetic (or the geometric) mean of these midpoints. If we adopt this approach, then in everyday lab practice we tend to exclude the first reversal points from the calculation (e.g., those gathered with the largest factor) and calculate the threshold with only the latest reversal points (i.e., those gathered with the smallest factor). In addition, Levitt (1971) recommends calculating the threshold on an even number of reversals in order to reduce estimation bias. This simple way of calculating the threshold of the participants has, however, some limitations. The most evident is that the threshold is calculated on a subset (and not all) of the trials run by the participant. It is possible to calculate the threshold or estimate the complete psychometric function of the participant (that is, the function representing the participant’s performance as a function of the stimulus level, for example, Δf) with other methods. These methods use all the trials run by the participant. For example, the experimenter may use the maximum likelihood approach (e.g., Green, 1990) or a Bayesian approach (Watson & Pelli, 1983). The Palamedes toolbox (Prins & Kingdom, 2018) and the Psignifit toolbox (Schütt et al., 2015) offer various functions to calculate the threshold using all trials. Notably, alternative ways of calculating the threshold make it possible to take into account behavioural biases that may exist in threshold estimation, such as when the participant prefers one response interval over the other (see Ulrich & Vorberg, 2009).

When we are estimating a threshold, how should we set the parameters of a staircase? There is a general trade-off in psychophysics: robust threshold estimation requires long-duration experiments, although recently there have been attempts to understand whether short experiments can gather reliable data, with positive responses (see Zhao et al., 2022; Mok et al., 2023). With this in mind, let us examine some typical parameters of psychoacoustic experiments. In *n*AFC tasks, the efficiency of the threshold tracking increases with the number of alternatives (Schlauch & Rose, 1990). However, because in audition alternatives are often delivered over various sound intervals (i.e., mI-*n*AFC), increasing the number of alternatives often coincides with increasing the duration of the trial and of the experiment (Schlauch & Rose, 1990). Typically, psychoacoustical research adopts the 2AFC or 3AFC tasks, and the number of intervals generally does not exceed three. The time/accuracy principle also applies to the variants of the transformed up-down methods. In particular, the transformed up-down methods are a good speed/accuracy trade-off when the two-down/one-up is used in combination with a 3AFC task, or when the three-down/one-up is used in combination with the 2AFC task. When using the transformed up-down rules, the number of reversals usually does not exceed 16, with at least four reversals run with a large factor, and the remaining run with a small factor. Experimenters may opt for a shorter experiment and gather 12 reversals, four with a large factor and eight with a small factor. Of course, even shorter experiments can be run, by reducing the number of reversals gathered during the threshold tracking. Once the characteristics of the threshold tracking are set, it is convenient to repeat the measurement of the threshold in several blocks of trials. A final recommendation is to favour the comfort of the participant: the starting delta of the experiment should be sufficiently high for an easy first set of trials. In addition, we recommend spending some time to introduce and familiarise the participant with the task. The participant has to understand well what they are asked to respond to. In many cases, poor performance can originate from simple misunderstanding. For example, in many languages, “high tone” can mean “high in pitch”, “high in loudness”, or simply “bright”, and this may generate confusion in the experiments, because “tell me the highest tone” becomes an ambiguous question (see Bruzzi et al., 2017; Pitteri et al. 2017; Pitteri et al., 2021). Therefore, it is good practice to spend a few minutes introducing the task to the participant, in particular when they are not expert listeners. PSYCHOACOUSTICS-WEB provides familiarisation trials before the experiment.

## Digital tools for auditory testing

This section provides an overview of major tools available for hearing testing, particularly those helping the experimenter in conducting experiments on auditory psychophysics. The section does not review music-related tools (e.g., Larrouy-Maestri et al., 2019) or tools dedicated to more “cognitive” auditory tasks (e.g., rating and sorting, Donhauser & Klein, 2023). Finally, we specifically focus on tools that are distributed with supporting documentation: although other tools exist (e.g., the National Institutes of Health [NIH] Toolbox Hearing Threshold Test or hearWHO, developed by the World Health Organization), they often provide limited information about the tool itself and appear unsuitable for research purposes.

The researcher interested in commencing a study in auditory psychophysics has a variety of software options available today. These tools unfold along two major dimensions. Firstly, tools can be generic, allowing the creation of any experiment type. Alternatively, they can be specific and dedicated solely to auditory psychophysics, with some offering a selection of “ready-to-use” experiments for auditory threshold estimation. The second dimension considers whether the tool is desktop or web-based software. One classic example of generic desktop software is Psychtoolbox (Brainard, 1997; Pelli, 1997) and its successor Psychtoolbox-3 (Kleiner et al., 2007). Psychtoolbox is a set of MATLAB functions that enable the creation of psychophysical experiments, including psychoacoustics experiments and threshold estimation. Psychtoolbox is free and also works under Octave, the free alternative to MATLAB. However, although the functions offered are numerous and extremely powerful and flexible, the use of the toolbox requires substantial programming skills. Grassi and Soranzo (Grassi & Soranzo, 2009; Soranzo & Grassi, 2014) wrote two desktop toolboxes (MLP and PSYCHOACOUSTICS) that enabled auditory threshold estimation for a wide variety of acoustical parameters and with a wide range of methods and algorithms (i.e., including the transformed up-down). The toolboxes come with a graphical interface and several built-in, classic psychoacoustics experiments ready to use at a mouse click. In addition, existing experiments can be customised, and with a few lines of MATLAB code, brand-new experiments can be created. However, the usability of these toolboxes is constrained by various limitations. The major one is that the toolboxes were written in MATLAB and working under a MATLAB environment. Occasional failure to work can be observed when MATLAB is updated and some functions are modified and/or deprecated. Last but not least, MATLAB is an expensive, proprietary software that limits the usability of the tool for researchers with scarce economic resources. Francart et al. (2008) developed APEX 3, a software test platform for auditory behavioural experiments. The software runs under a Windows environment. APEX 3 provides the user the ability to create experiments from scratch, including adaptive procedures, by writing XML files that include the various steps of an experiment. A similar idea was developed in PsyAcoustX by Bidelman et al. (2015). This suite is written in MATLAB. PsyAcoustX consists of several modules that enable users to build custom auditory experiments. The modules include the transformed up-down methods of Levitt (1971). In more recent years, Sek and Moore (2020, 2021) have developed PSYCHOACOUSTICS, a Windows-based software that, similarly to MLP and PSYCHOACOUSTICS by Soranzo and Grassi (2014), allows one to set up and conduct a wide range of psychoacoustic experiments. Sek and Moore’s PSYCHOACOUSTICS requires no programming skills and offers several ready-to-use experiments that implement the transformed up-down methods. The main limitation of this toolbox seems to be flexibility: existing experiments can be customized but new experiments cannot be created. In addition, the toolbox—like APEX 3—must be implemented in the Windows operating system. Finally, PART (Portable Automated Rapid Testing; Gallun et al., 2018) is an app-based tool that is accessible by tablets and smartphones (i.e., not by desktop PC), which makes the tool adept for remote testing. PART includes a wide array of ready-to-use psychoacoustical tests. In the current version, when an experiment is run, data are stored in the mobile device itself. The app-based nature of the software, however, limits its connectivity with good hardware audio equipment, such as external sound cards or attenuators, and it is thus not optimal for laboratory testing. The last few years have seen the growth and diffusion of web-based tools. Among generic tools, JSPSYCH (De Leeuw, 2015), when combined with specific plugins dedicated to psychophysics (Kuroki, 2021), is the web-based counterpart of Psychtoolbox-3. However, once again, the tool requires substantial programming skills (i.e., JavaScript). More recently, Sulas et al. (2022) developed a set of features for the open-source Python-based OpenSesame platform that allows the creation of custom behavioural and cognitive hearing science tests. These features exploit the graphical interface of OpenSesame (Mathôt et al., 2012) and do not require programming skills (but mastering OpenSesame is of course necessary). All web-based tools, like any web application, enable remote testing by sharing the experiments via direct links.

As the reader may have already inferred, tools are many, various and different, and there is no single best (or worst) tool. Overall, generic tools offer flexibility and customizability at the cost of a relatively long learning phase. Some learning is also necessary in specialised applications that offer modules for the creation of experiments. In contrast, specific tools with built-in experiments are usually fast to learn but offer limited flexibility. Desktop applications provide better timing than web-based tools (Bridges et al., 2020), but this factor has little influence in auditory psychophysics (whereas it is vital in visual psychophysics). Desktop tools are often written under a proprietary environment (usually MATLAB), and this is a major limitation: the tool may stop working if the functions of the environment are deprecated, changed, or updated. In addition, the cost of the environment may negate the free accessibility of the tool.

PSYCHOACOUSTICS-WEB is a web-based tool specifically dedicated to auditory psychophysics. The current version of the toolbox comes with six ready-to-use classic experiments. PSYCHOACOUSTICS-WEB enables individual testing, in the lab or remotely. It can be used via PC, tablet, or smartphone, making the tool adept for research but also for teaching and dissemination (e.g., a public demonstration). Experimenter accounts can be created and experiments shared with remote participants via direct links. In addition (and this is perhaps a unique feature of PSYCHOACOUSTICS-WEB in comparison to available tools), PSYCHOACOUSTICS-WEB centralises the data archiving. Users can download data locally, in their own computer, but data are also saved in the server hosting the toolbox and located at the University of Padova, Department of General Psychology. This opens the possibility of creating a single, common database of auditory skills. One final note that is relevant for all tools including PSYCHOACOUSTICS-WEB: no tool can be a lifelong companion. Programming languages change over the years, and the language we are currently using may no longer exist tomorrow. In the classic paper by Lieberman and Pentland (1982), the authors provided the full code to implement the “best PEST”, a specific algorithm for sensory threshold estimation. However, because the software was written in BASIC, it is now virtually unusable. Table 1 provides a synthetic view of the various tools described in this section.

**Table 1:** Synthetic description of the digital tools that researchers can use to run classic experiments in auditory psychophysics

Name of the tool	Type	Use	Environment	Built-in experiments	Requires programming skills	Cost	Enables centralised data collection
Psychtoolbox-3 (Kleiner et al., 2007)	Generic	Desktop	MATLAB or Octave	No	Yes	Free, but the environment may have a cost	No
MLP and PSYCHOACOUSTICS (Grassi & Soranzo 2009; Soranzo & Grassi 2014)	Specific	Desktop	MATLAB	Yes	No (minimal if the user aims to create brand-new experiments)	Free, but the environment has a cost	No
APEX 3 (Francart et al., 2008)	Specific	Desktop	Windows	No	Minimal	Free	No
PSYCHOACOUSTICS (Sęk & Moore, 2020, 2021)	Specific	Desktop	Windows	Yes	No	Free, but the user manual is sold separately	No
PsyAcoustX (Bidelman et al., 2015)	Specific	Desktop	MATLAB	No	No	Free, but the environment has a cost	No
PART (Gallun et al., 2018)	Specific	Tablets and smartphone only		Yes	No	Free	No
Psychophysics plugin for JSPSYCH (Kuroki, 2021)	Generic	Web-based	JSPSYCH	No	Yes	Free	Potentially yes
Sulas et al. (2022)	Specific	Web-Based	OpenSesame	No	No, but the knowledge of OpenSesame is required	Free	Potentially yes
PSYCHOACOUSTICS-WEB	Specific	Web-based	Stand-alone	Yes	No	Free	Yes and implemented

## PSYCHOACOUSTICS-WEB

PSYCHOACHOUSTICS-WEB can be accessed through the following link: http://psychoacoustics.dpg.psy.unipd.it/sito/index.php .

**Fig. 1:**
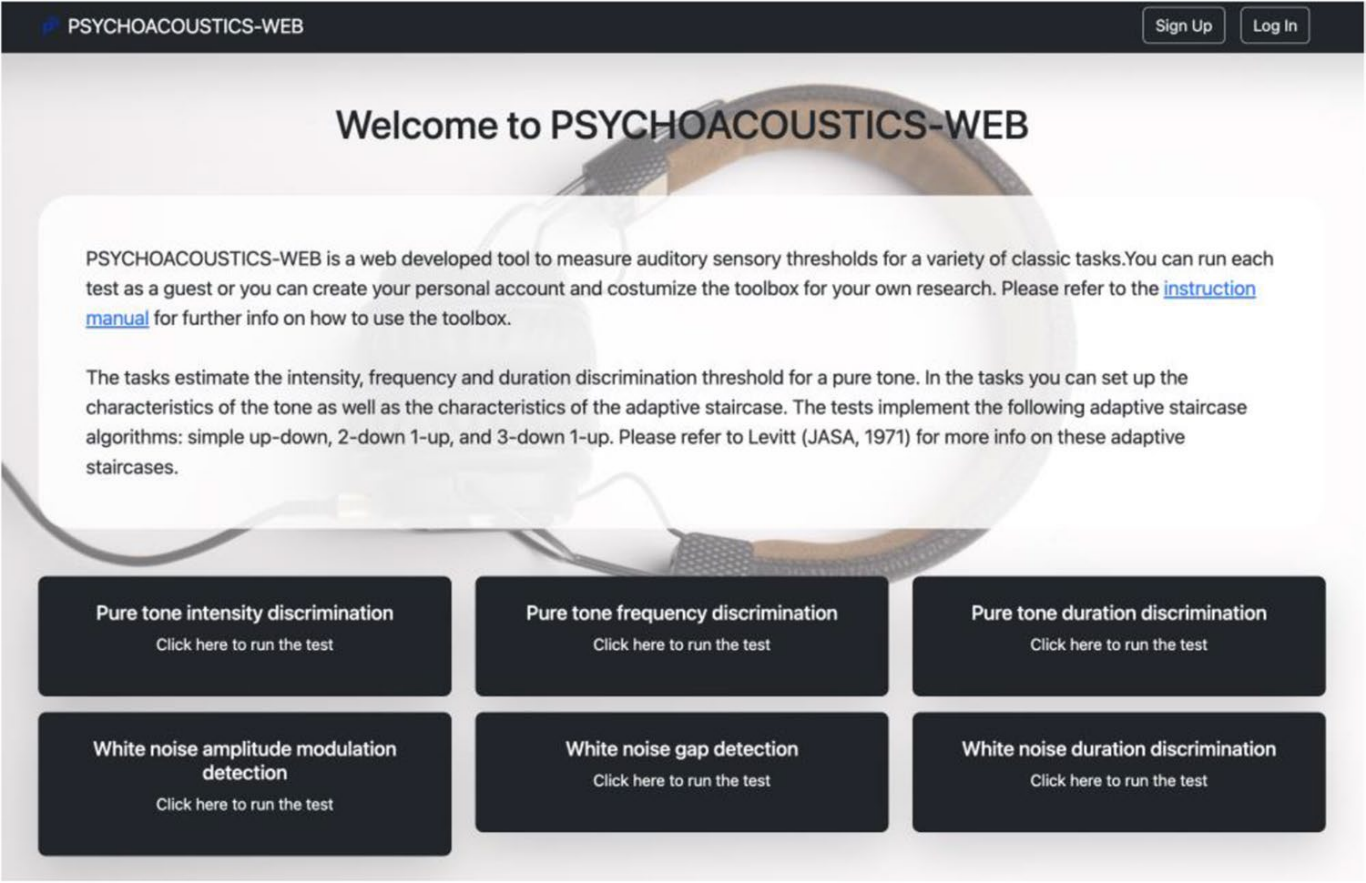
Landing webpage of PSYCHOACOUSTICS-WEB. *Note.* Interface of PSYCHOACOUSTICS-WEB. When the user lands on the home page, they can select and run a particular experiment, retrieve the instruction manual, log in to the personal account, or sign up to create a personal account

There, the user finds the home page of the toolbox (Fig. 1). On the home page, the user can see a welcome text, the list of the available experiments, a link to the manual, and the terms and conditions. Manual and terms and conditions may be updated, and we suggest referring to the most up-to-date information by checking them regularly. The toolbox was created with two major types of users in mind: an occasional user willing to take one experiment autonomously, and an experimenter using the toolbox for their own research. The experimenter can also have a further type of user, the participant. We firstly describe the toolbox from the point of view of the occasional user. Later, details will be given for the experimenter and the participants of the experimenter.

**Fig. 2:**
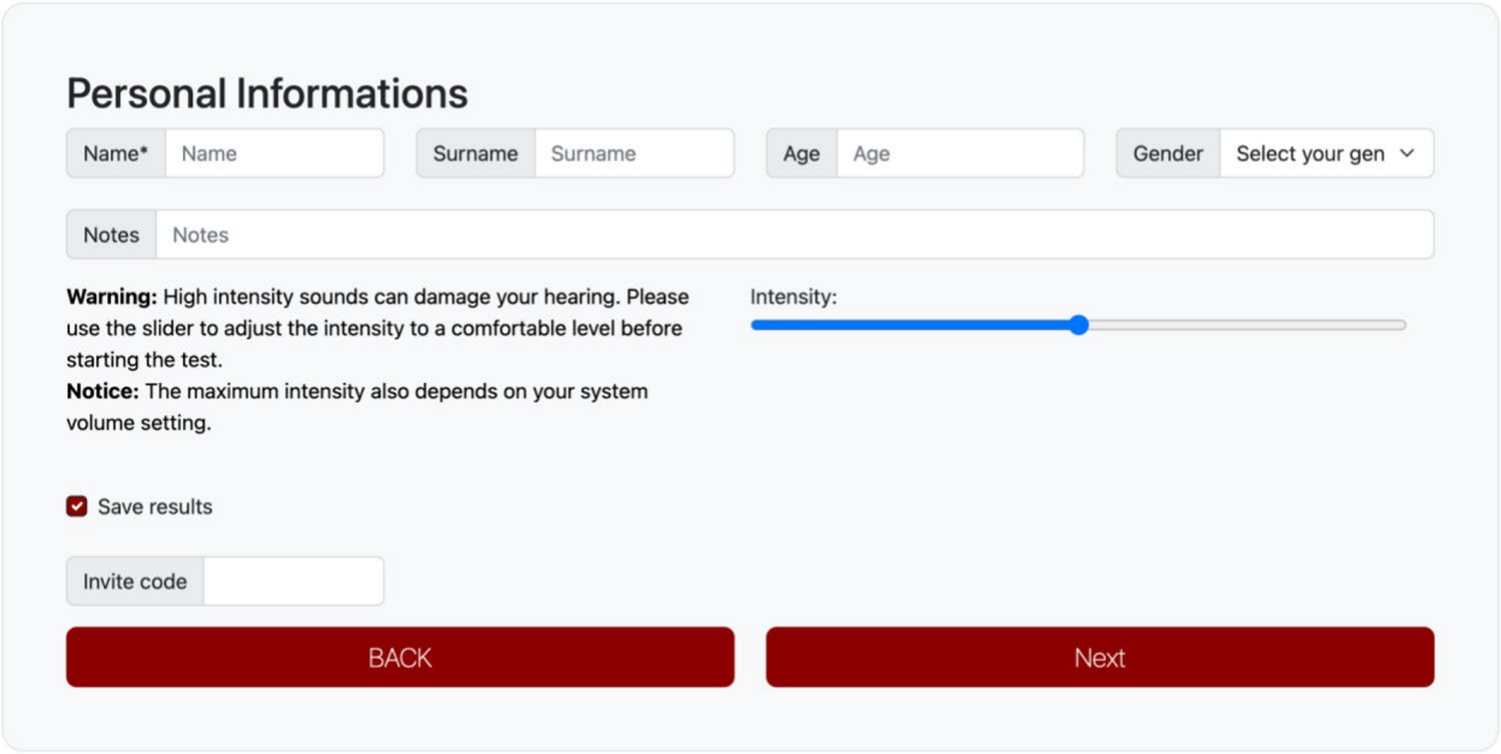
The participant’s details. *Note*. Interface of PSYCHOACOUSTICS-WEB. Here the user is asked to provide their personal details

The first thing the occasional user must do is select the experiment they want to run. After that, they are prompted to a page in which they have to input name, surname, age, and biological sex (see Fig. 2). These fields also enable one to identify the user’s data in the server’s database. In addition, there is a free-text field in which the user can type custom information about the session (e.g., whether they have any hearing impairment) and a volume adjuster that plays a sample sound and enables the user to set the volume at a comfortable level. The “invite code” will be discussed in detail later. It is an alphanumeric code that the user may have received from another user (e.g., an experimenter) that will prompt the occasional user to a specific experiment with specific characteristics selected by this user.

**Fig. 3:**
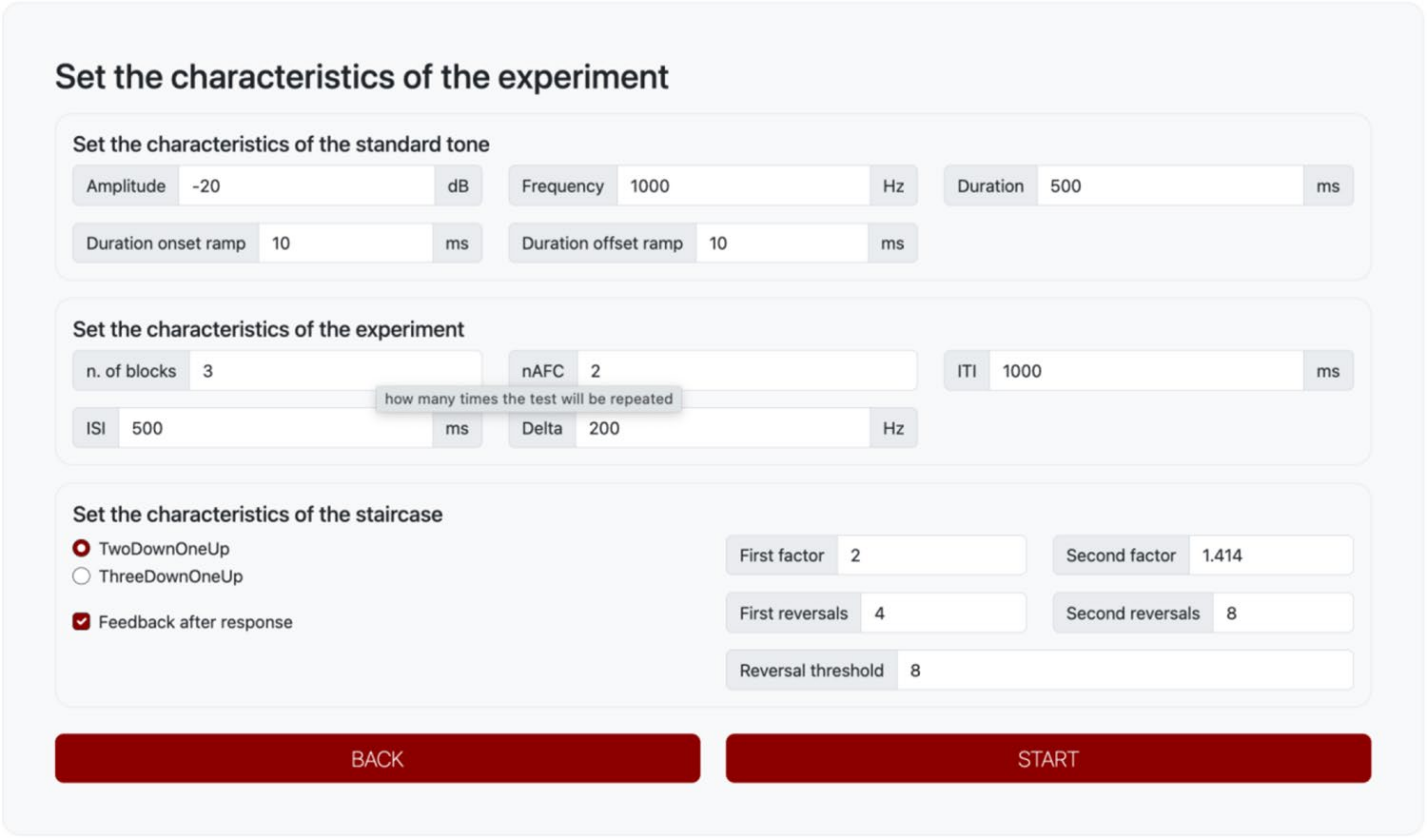
Page to set the characteristics of the experiment. *Note*. Main page of the toolbox in which the participant can set the characteristics of the experiment, stimuli presented, and the threshold tracking. In the case in the figure, the experiment is a frequency discrimination threshold. The starting delta is 200 Hz, and the experiment will run three blocks of trials with a 2IFC task. The two-down/one-up staircase will be used for threshold tracking. For the first four reversals, delta will be changed with a factor 2, and for the next eight reversals, delta will be changed with a factor $\sqrt{2}$. Threshold will be calculated on the last eight reversals

After clicking “next”, the user is prompted to the graphical interface that controls the characteristics of the experiment (see Fig. 3). At the top of the page, the user can change the characteristics of the standard stimulus. For example, for sine waves, the user can set/change the amplitude (in decibels relative to full scale [dB FS]), the frequency (in hertz) and the duration (in milliseconds) of the standard tone (the frequency parameter is missing for the noise carriers). They can also change the duration of the onset/offset raised cosine ramps that modulate the amplitude of the beginning/end of the tone. In the experiment, the characteristics of the variable tone will be identical to those of the standard, except for the characteristic that is manipulated in the experiment (e.g., the frequency of the variable tone for the frequency discrimination threshold experiment). In the middle of the page, the user can set/change the key parameters in the experiment. These parameters include the number of blocks of trials, the alternatives/intervals of the forced-choice procedure, the duration of the inter-trial interval (ITI), the duration of the inter-stimulus (ISI), and delta. ITI is the time (in milliseconds) between the completion of one trial and the commencement of the next. ISI is the time (in milliseconds) separating the stimuli within one trial. Delta is the parameter that varies during the experiment as a function of the participant’s response. Here, the user sets its first value. In frequency, duration, and intensity discrimination tasks, delta is the starting difference (in frequency, duration, and sound pressure level) between the standard and variable. In gap detection tasks, delta is the starting duration of the gap, whereas in amplitude modulation detection tasks, delta is the starting percent of modulation of the modulated noise.

At the bottom of the page, users can select the adaptive procedure they wish to use. PSYCHOACOUSTICS-WEB offers two options: the two-down/one-up and the three-down/one-up. Here, the user can set the value of the “factor”, one for the first *N* reversals, the other for the successive reversals. Default factors are 2 and $\sqrt{2}$. The “reversal threshold” field sets the number of reversals that are included in the calculation of the threshold starting from the last reversal of the threshold tracking (see previous section for further details about this type of threshold calculation). For example, if the user sets four reversals with factor 2 and eight reversals with factor $\sqrt{2}$ and the reversal threshold is set to 6, the toolbox calculates the threshold on the last six reversals, the last six run with the factor $\sqrt{2}$.

When the user clicks “start”, the experiment begins. During the experiment the participant can respond indifferently with the keyboard, the mouse, or touchscreen. The various messages prompted to the user during the experiment are sufficiently straightforward. The software provides feedback after the response of each trial if the corresponding option was flagged on the graphical interface. At the end of the blocks, the user can see the threshold (i.e., the average to the thresholds calculated in each block) printed on the screen, and they can also download two text files: a csv text file including the individual thresholds of each block and an extended csv text file including a trial-by-trial log of the experiment.

**Fig. 4:**
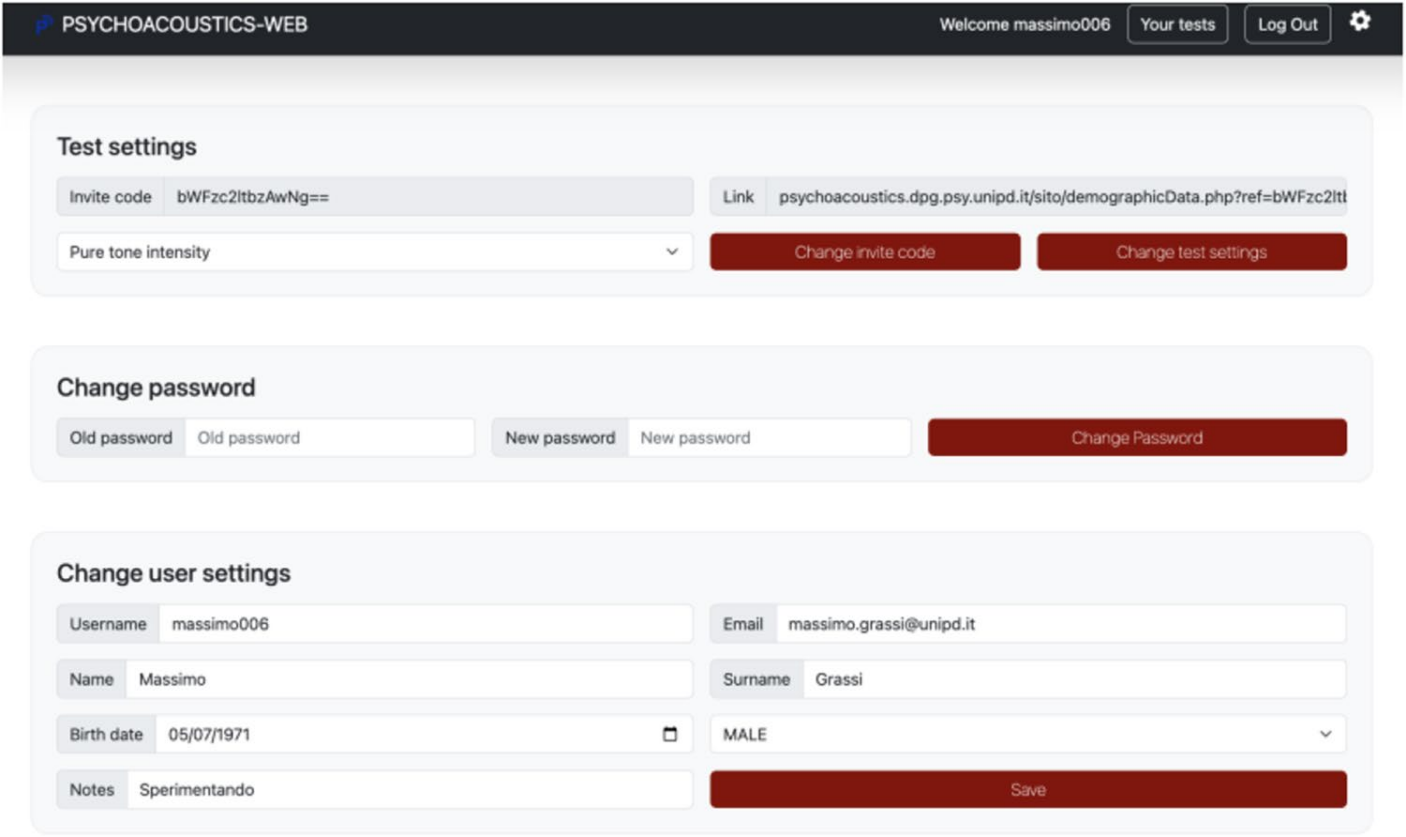
Interface that enables the user to manage their settings. *Note*. This is the page the user sees after logging in to the toolbox and clicking on the gear wheel at the top right of the page

The toolbox was designed to be used for research as well. The user can create a personal account, and personal accounts were designed to be suitable for experimenters using the toolbox for their own research (see Fig. 4). For example, with the personal account, the user (from now on simply the “experimenter”) can run “draft” experiments (e.g., when selecting the specific values of the parameters for an experiment) and later, when parameters are set, send the experiment to further users (e.g., the participants of an experiment) in the form of a direct link.

At the home page of the toolbox the experimenter can sign up and create a personal account. When the account is created, the experimenter can log in to the toolbox. If the experimenter logs in, the top right corner of the home page looks different from that of an occasional user. By clicking the gear wheel, the experimenter now has access to various new fields and information (see Fig. 4).

Here the experimenter can change the password and update their personal details (middle and bottom part of the page). At the top, the experimenter can select one of the experiments of the toolbox and, after clicking on “change test settings”, change the parameters of the selected experiment to their own needs. After clicking, the experimenter is prompted to the parameters page of the desired experiment. There, they can set and save the parameters of the selected experiment. Once this is done, the experimenter can return to the login page. The parameters will be stored until the experimenter revisits the page to make changes. On the login page, there is a link and an invite code. The link is a direct link to the experiment customised by the experimenter, in other words, a link that enables another user (e.g., a participant of the experiment) to access the experiment directly with the settings determined by the experimenter. The invite code serves the same purpose as the link: if passed to a user and inputted in the appropriate field, it prompts the user to the experiment, with the settings determined by the experimenter.

**Fig. 5:**
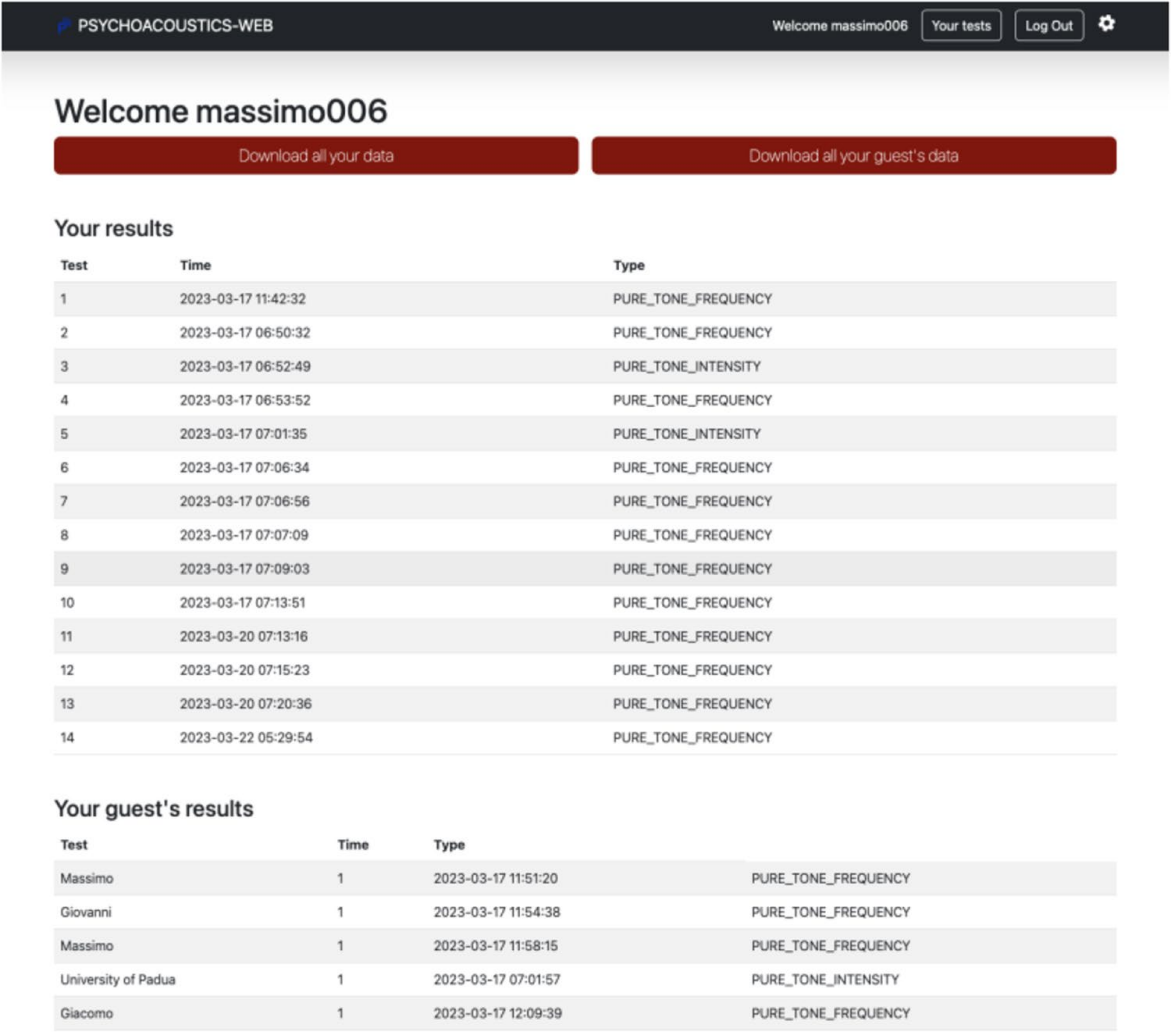
Data interface. *Note.* Results page in the experimenter account which also includes data collected from remote participants

When the experimenter clicks on “your tests”, they are prompted on the data (see Fig. 5). Here the experimenter can see at the top of the page the results and the data for the experiment run by their own account (e.g., the pilot experiments run before the proper experiment) and, at the bottom of the page, the data of the guests that participated in their experiments via direct link or invite code (i.e., the participants). Here the experimenter can download the data. The data are written into a csv text file.

The toolbox provides two distinct types of datafiles: a reduced datafile and a complete datafile. The reduced datafile includes the listener’s demographic details and threshold estimates for each block of trials. In contrast, the complete datafile serves as a comprehensive log that encapsulates all facets of the experiment. It includes the experiment's name, the participant’s demographics, the standard tone characteristics, the characteristics of the threshold tracking, and a comprehensive event log for each individual trial. Both datafiles are stored in CSV text format and are structured as square matrices, characterised by rows and columns. The final datafile comprises various columns, some of which are as follows:

- *n*AFC (*n*-alternative forced choice): reports the number of choices available in each trial
- ISI (inter-stimulus interval): reports the interval stimulus duration
- ITI (inter-trial interval): reports the inter-trial interval duration
- Reversal threshold: reports the number of reversals that are used to calculate the threshold
- Algorithm: reports the algorithm used in the experiment
- Delta: reports the trial-by-trial value of the delta parameter
- Correct: reports whether the response in a trial was correct or not
- Reversals: reports the trial-by-trial number count of the reversals accumulated thus far

All the fields of the header of the datafiles are described in the Appendix 3. These datafiles (in particular the extended one) are a comprehensive and detailed documentation of the experiment. They facilitate a thorough analysis and interpretation of the results and enable the calculation of the threshold in various ways (e.g., (Prins & Kingdom, 2018; Schütt et al., 2015; Ulrich & Vorberg, 2009). For further information, please refer to the manual.

### Testing of the toolbox

The toolbox underwent testing in two different contexts: in the laboratory and in a public setting. In the laboratory, a group of listeners was asked to perform a task twice. One implementation of the task used a classic toolbox (PSYCHOACOUSTICS, Soranzo & Grassi, 2014), while the other implementation used the web-based version, PSYCHOACOUSTICS-WEB. In the public setting, PSYCHOACOUSTICS-WEB was tested at a science exhibition dedicated to human senses, and the results were compared with those in the literature.

In the laboratory, 24 listeners (13 female, age range 19–51 years) were asked to participate in an experiment estimating the frequency discrimination threshold for a 1-kHz pure tone. Listeners reported normal hearing and were recruited among colleagues and students still in the laboratory in the hot month of July 2023. They signed an informed consent form for the participation. The experiment was conducted in a single-walled IAC soundproof booth. Sounds were produced with a desktop PC connected to a Focusrite Scarlett 4i4 sound card. The output of the soundcard was connected to a pair of Sennheiser HDA 300 headphones. Listeners received an oral introduction to the experiment and were asked to run six familiarisation trials before starting the experiment. In these familiarisation trials, the listener listened to three 200-ms sine tones separated by a 500-ms silent interval. Two tones were identical whereas the third tone (randomly the first, the second, or the third of the sequence) was of a higher frequency (100 Hz or 50 Hz). The listener was asked to report the higher-pitch tone. When the listener responded correctly to a block of six consecutive trials, the experiment started. The experiment was divided into eight blocks of trials; half of the participants took four blocks with PSYCHOACOUSTICS followed by four blocks with PSYCHOACOUSTICS-WEB. For the remaining participants the order was reversed. Both software programs offered the same experiment. In both we set a two-down/one-up staircase method with two reversals made with a factor 2 and six reversals made with a factor $\sqrt{2}$. The trial was a 3I-3AFC task, and each block tracked the 70.7% threshold. The frequency difference presented at the first trial of each block of trials was 100 Hz. In the following trials the difference was varied following the staircase algorithm as a function of the listener’s response. The threshold was the average of the deltas registered at the last six reversals of each block of trials.

**Fig. 6:**
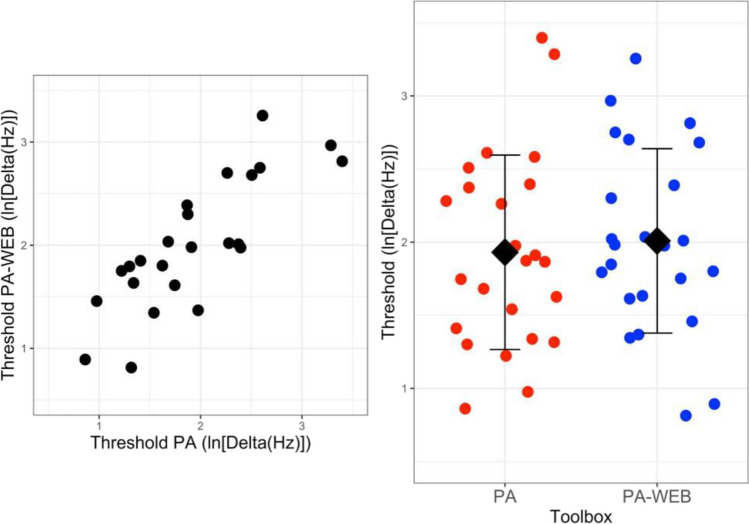
Lab comparison between PSYCHOACOUSTICS (Soranzo & Grassi, 2014) and PSYCHOACOUSTICS-WEB. *Note.* On the left, individual thresholds estimated with PSYCHOACOUSTICS (PA in the plot) plotted against those collected with PSYCHOACOUSTICS-WEB (PA-WEB in the plot). On the right, individual thresholds estimated with PSYCHOACOUSTICS (PA, left) and PSYCHOACOUSTICS-WEB (PA-WEB, right). The graph also shows the mean and the standard deviations of the data

For each listener, the four thresholds collected with PSYCHOACOUSTICS and the four thresholds collected with PSYCHOACOUSTICS-WEB were averaged and the natural logarithm of both averages was calculated. The log-thresholds collected with PSYCHOACOUSTICS and PSYCHOACOUSTICS-WEB were compared with a paired *t*-test. The thresholds were not different, and the Bayes factor favoured the null hypothesis, *t*(23) = 0.38, *p* = .71, *d* = 0.11, BF = 0.30. The correlation between the two threshold estimates was high: *r*(24) = 0.81. Thresholds are presented in Fig. 6.

The toolbox was also tested in a science exhibition (“Sperimentando2023”, https://sperimentandoaps.wordpress.com/sperimentando-2023/). “Sperimentando” is an exhibition for middle and high school students but is also open to the public. Each year the exhibition is dedicated to a different science theme, and the 2023 edition was dedicated to the human senses. PSYCHOACOUSTICS-WEB was offered in the part of the exhibition dedicated to hearing. Here, visitors received information about human hearing and human hearing testing via two posters hanging on the wall. In addition, visitors could test their sensitivity for acoustic frequency via one experiment created with PSYCHOACOUSTICS-WEB. They could take the test with a desktop PC available on-site or with their mobile devices. A URL link was created for the experiment (see Fig. 4, top right). The experiment could be accessed by the mobile device via a QRcode that pointed to the link. Written instructions informed the visitors about the testing, its approximate duration (about 2 minutes), and how to interpret the score returned by the software. A table depicted the reference thresholds for a hypothetical population divided into deciles. The values in this table were those reported by Kidd et al. (2007), rounded to provide numbers “easy to read” by the visitors. Visitors were also advised to wear headphones during the experiment.

The experiment was a frequency discrimination threshold implemented via two-down/one-up adaptive tracking and a 3I-3AFC task. This staircase tracks 70.7% of the psychometric function. In order to obtain a quick threshold measurement, the staircase ran only six reversals; the first two reversals were run with a factor 2, the remaining four reversals were run with a factor $\sqrt{2}$. The threshold for the participant was calculated by averaging the threshold estimate at the last four reversals. The experiment included only one block of trials. The standard tone was a 250-ms, 1-kHz sine wave gated on and off with two 10-ms raised cosine ramps. In the first trial of the block of trials, the difference between standard and variable (i.e., the delta) was 100 Hz (a difference of about 1.5 semitones in musical terms). For this particular implementation, in order to guarantee the anonymity of the participants, they were advised to use a nickname, and demographic details such as age and sex were not required: participants could provide them if they wanted to. Participation in the experiment was voluntary. Data were collected throughout the duration of the exhibition (i.e., from 13 April to 14 May 2023). During this time the experiment was completed by 120 unique visitors. This sample was composed of 54 female and 51 male participants (35 participants did not provide information about their biological sex). Forty participants did not disclose their age and one participant provided an age that seemed highly improbable (i.e., 1 year old). The average age of the remaining participants was 29 years, ranging from 11 to 77 years. Thresholds higher than 50 Hz (about one musical semitone) were removed from the analysis. The remaining 96 thresholds were divided into deciles and compared with those collected by Kidd et al. (2007). In Table 2, thresholds collected during “Sperimentando” and divided into deciles are compared with the deciles of Kidd et al. (2007).

**Table 2:** Comparison between the thresholds obtained by Kidd et al. (2007) and those obtained with PSYCHOACOUSTICS-WEB during the “Sperimentando” exhibition (see text). The table reports the threshold estimate for each decile. The middle column reports the absolute value of the difference between the two values of each row, and the last two columns on the right report the natural logarithm of the thresholds reported in the first two columns

Decile	PSYCHOACOUSTICS-WEB - threshold (Hz)	Kidd et al. (2007) - threshold (Hz)	|Difference| (Hz)	PSYCHOACOUSTICS-WEB - natural logarithm [ln(Hz)]	Kidd et al. (2007) - natural logarithm [ln(Hz)]
1 (best)	5.17	3.08	2.09	1.64	1.12
2	6.44	4.95	1.49	1.86	1.6
3	7.54	6.59	0.95	2.02	1.89
4	9.75	7.88	1.87	2.28	2.06
5	11.32	8.78	2.54	2.43	2.17
6	14.11	10.96	3.15	2.65	2.39
7	15.12	12.55	2.57	2.72	2.53
8	21.35	13.99	7.36	3.06	2.64
9	29.54	15.45	14.09	3.39	2.74
10 (worst)	45.27	36.27	9	3.81	3.59

*Note.* The table reports the thresholds interpolated by Kidd et al. (2007, see Table 3, first row). In that study, the authors recruited 340 participants and asked them to complete several auditory tasks, including a frequency discrimination task with pure tones

A visual inspection of the table reveals that the performance of listeners collected during “Sperimentando” and that collected by Kidd et al. (2007) are very similar. The thresholds collected during “Sperimentando” are higher, but this is not surprising, for two reasons: (i) the noisier conditions of the testing during “Sperimentando” in comparison to the laboratory conditions of Kidd et al. (2007), and (ii) the longer testing of the latter study, which collected 72 trials per participant, about double the number of trials collected during “Sperimentando” for each listener. “Sperimentando” returned yet another important piece of information: with PSYCHOACOUSTICS-WEB we did not receive any feedback regarding issues, regardless of the type of device, internet connection, operating system, or browser. In summary, both empirical tests of PSYCHOACOUSTICS-WEB provided solid evidence of the tool's reliability and robustness.

### Information for users

This section is dedicated to colleagues that may potentially use the toolbox for their own research either in the laboratory or remotely. The information included here may be trivial to people working in the auditory field, but we think it is important and relevant for those colleagues that may come from different fields and may use the toolbox as a side tool for an experiment including multiple tasks.

The first problem the user may come across is the selection of the experiment. The current version of PSYCHOACOUSTICS-WEB offers six tests to explore diverse facets of human auditory perception; these tests serve to evaluate auditory skills along various sound domains. These domains emerged in the comprehensive study by Kidd et al. (2007) that administered 19 auditory tasks to a large group of listeners. Tasks presented synthetic sounds (i.e., pure tones, broadband noise) and recorded sounds (i.e., speech sounds and environmental sounds), each exploring various dimensions of sound perception. The study revealed that the perception of synthetic sounds unfolds along three dimensions: “loudness and duration”, “amplitude modulation”, and “pitch and time”. These dimensions are represented in the tests implemented in PSYCHOACOUSTICS-WEB. The pure tone frequency discrimination measures the frequency discrimination threshold of pure tones and it is valuable for assessing the sensitivity of listeners for the frequency domain and represents the “pitch and time” dimension. The pure tone duration discrimination, the duration discrimination (noise carrier) and the pure tone intensity discrimination measure, respectively, the duration discrimination threshold of pure tones and noise, and the intensity discrimination threshold of pure tones offer insights into the “loudness and duration” dimension. The gap detection and the amplitude modulation detection measure the ability to detect changes in the temporal envelope of a sound and represent the “amplitude modulation” dimension.

When running these experiments, the experimenter may ask whether the threshold estimate is veridical or is (for example) an outlier. There are various literature works that can aid the reader in evaluating the threshold estimates gathered with the toolbox. The very same work by Kidd et al. (2007) provides reference values to evaluate the measures returned by the toolbox. Note that some individual differences may change (often greatly) the performance of a listener in an auditory task. A key factor is of course the hearing health of the listener: people with a hearing impairment (any kind and origin) have worse performance than normal-hearing people (e.g., Lentz et al., 2022). Another—and correlated—factor is age. Young adults tend to perform better than older adults in any auditory task (Grassi & Borella, 2013). A certain familiarity with sounds may be yet another factor. For example, musicians often have better auditory performance than nonmusicians (Micheyl et al., 2006; Rammsayer & Altenmüller, 2006), an advantage that persists over the life span (Zendel & Alain, 2012; Grassi et al., 2017). Occasionally, some individuals may perform poorly in selected auditory tasks. For example, people suffering from congenital amusia (Peretz, 2016) show selective difficulty in frequency discrimination tasks (Hyde & Peretz, 2004). However, congenital amusia is rare in the population (about 1.5% of the population).

The quality of the sound produced by the toolbox is exclusively dependent on the hardware of the user, namely, the sound card and the headphones (or other type of equipment) that converts the digital sound into the acoustic wave delivered to the ears of the participant. In particular, the sample rate of the sounds generated by the toolbox is the default sample rate of the browser. In our testing, we never observed a sample rate lower than 44,100 Hz (the standard for audio CDs), and the largest share of browsers seem to work at 48,000 Hz (i.e., quality higher than the standard audio CD).

One important factor that the user needs to keep in mind is that the toolbox does not enable one to control the absolute intensity of sounds, but only the relative intensity. This is true for all toolboxes listed in Table 1, and it is also why no toolbox offers an estimate of the absolute threshold (i.e., the minimum audible intensity), which is an auditory dimension that is often looked for by scientists willing to assess the hearing of their participants. The absolute intensity of the sounds at the listener’s ear depends on the hardware (sound card and headphones). The intensity the user can set via the PSYCHOACOUSTICS-WEB interface is in dB FS (see “dB FS” in Wikipedia for a further explanation), a decibel scale in which 0 is assigned to the maximum possible *digital* level, and negative infinity to the lowest possible *digital* intensity. It is possible to calibrate the toolbox so that the sounds are delivered at a known intensity. The calibration requires external hardware (the so-called artificial ear) that has to be coupled to the sound-output device. The specific calibration hardware depends on the specific sound-output device (e.g., circum-aural, supra-aural or in-ear). When calibrating, it is important to fix (or know) the various “volume” levels of the apparatus because they determine the output level of the apparatus. For example, currently PSYCHOACOUSTICS-WEB enables one to control the intensity of the stimuli in two places: in the graphical interface, where the parameter for the experiment can be set, but also via the “volume adjuster” that is presented to the listener to adjust the sound output (if necessary) at a comfortable listening level. Note that the operating system has its own volume, and the sound card (if external) may have its own volume control. In practice, when calibrating, the experimenter needs to take into account all of these volume controls.

If we are using PSYCHOACOUSTICS-WEB for online testing we will face different types of issues. A fist problem we may come across is the noise level of the environment in which the test takes place. Usually, psychoacoustic experiments are performed in silent environments such as soundproof booths. PSYCHOACOUSTICS-WAB has currently no tool to measure the noise level of the environment in which the participant is taking the test. If the experimenter is worried about the noise level of the environment, they should know that there are several mobile applications that enable the measurement of the noise level with a smartphone. The measures returned by these apps are sufficiently accurate for an experimenter willing to have an estimate of the noise level of the environment, and a noise-level below 45 dBA is definitely sufficient for auditory testing^1^.

Another problem, yet again related to online testing, is whether the participant is using headphones, earphones, or speakers of any kind. Typically, headphones are preferred for auditory testing because they enable the participant to better isolate acoustically from the environment (some headphones provide very good sound isolation from the environment). There is currently no way to know, via software, whether the user is using headphones or, for example, loudspeakers. However, there are several behavioural solutions that help in screening whether a participant is wearing headphones or earphones or listening through loudspeakers (Woods et al., 2017; Milne et al., 2021; Wycisk et al., 2023a, 2023b). These screenings make use of test sounds that can be perceived only with headphones. For example, Milne et al.’s (2021) screening plays Huggins pitch stimuli to listeners. The Huggins pitch stimulus is an illusory pitch phenomenon generated by two slightly different white noise samples that are delivered to the left and the right ear. When the stimulus is listened to via headphones, the listeners can perceive a faint tonal object embedded in noise. This screening test correctly detects 80% of headphone users and has a false-positive rate of 20%. The codes for implementing this screening are publicly available in JavaScript and through Gorilla.

When tests are conducted remotely, there is no possibility of having an absolute estimate of the sound intensity that is presented at the level of the listener’s ears. In addition, because each listener can set their own volume settings autonomously, we may end up with stimuli that are presented at different levels. Recent studies have shed some light on the possibility of conducting auditory psychophysics remotely even without knowing the sound intensity the listener is listening to. First of all, not all auditory tasks require precise knowledge of the intensity level. In general, tasks tapping the frequency and temporal domains are relatively unaffected by the presentation level. Mok et al. (2023) conducted several auditory tasks with various cohorts of listeners recruited online and compared web results with identical lab experiments. The results were almost identical, with negligible differences in effect size. Zhao et al. (2022) investigated whether it was possible to conduct intensity-dependent tasks such as the detection of a tone signal presented in a band of noise, in other words, tasks in which intensity differences may reverberate in differences in the results. The authors first measured the absolute threshold of the participant with noise whose digital amplitude was known. Participants tracked this absolute threshold by adjusting the volume of the operating system, and they were subsequently asked to fix the system volume at this threshold value. Then, the tone-in-noise experiment presented a digital noise whose digital amplitude was 40 dB higher than the noise used to estimate the absolute threshold. Note that both Mok et al. (2023) and Zhao et al. (2022) dedicated a consistent amount of effort in screening participants for various parameters (use of headphones, possible audiometric hearing loss, etc.); a good selection of participants seems an important prerequisite for good auditory online testing.

Another possible issue related to our tool may be the specific browser used for the experiment. So far, the toolbox has had no issues with common browsers available on the market: Chrome, Firefox, Edge, and Safari. We are not aware of issues with tablet or smartphone browsers. However, because of the great many browsers, operating systems, and devices and the constant updating they undergo, we cannot guarantee the functionality of the tool for any existing browser, environment, or device. If the user experiences an issue with a specific browser, operating system, or device, we suggest simply changing the browser and/or the operating system and/or the device. One last note for the reader: the toolbox is continuously updated (e.g., new experiments will hopefully be added and existing functionalities improved), and we encourage readers to give us suggestions and feedback on directions for improvement.

## Data Availability

Data and scripts are available in the Open Science Framework (https://osf.io/va8mp/) and GitHub (https://github.com/hurxan/psychoacoustics-web).

## Appendix

**Table 3:** The header of the datafiles returned by the toolbox. For each column header, it is explained the content of the column

Header field name	Content of the variable
Name	Name of the participant
Surname	Surname of the participant
Age	Age of the participant
Gender	Gender of the participant
Test count	An integer (from 1 to 6) that identifies each experiment currently implemented in the toolbox
Test type	The extended name of the experiment
Timestamp	The date/time when the datum was recorded
Sample rate	The sample rate of the sound delivered to the participant. It depends on the device.
Device info	A text providing information on the device, operating system, and browser
Amplitude	Amplitude (in dB FS) of the standard stimulus and the variable stimulus (if the variable stimulus is not varied in intensity)
Frequency	Frequency (in Hz) of the standard stimulus and the variable stimulus (if the variable stimulus is not varied in frequency). The field is empty if the stimulus is noise.
Duration	Duration (in ms) of the standard stimulus and the variable stimulus (if the variable stimulus is not varied in duration)
Onset ramp	Duration of the onset ramp of the standard and variable stimulus (in ms).
Offset ramp	Duration of the offset ramp of the standard and variable stimulus (in ms)
Modulator amplitude	Modulation amplitude (in dB) of the modulator that is applied to the noise carrier in the amplitude modulation detection experiment. The field is empty if the user is running a different experiment.
Modulator frequency	Modulation frequency (in Hz) of the modulator that is applied to the noise carrier in the amplitude modulation detection experiment. The field is empty if the user is running a different experiment.
Modulator phase	Phase (in radians) of the modulator that is applied to the noise carrier in the amplitude modulation detection experiment. The field is empty if the user is running a different experiment.
Number of blocks	Number of blocks of trials set by the user
*n*AFC	Number of forced alternatives set by the user. Note that in the current version of the toolbox, the number of alternatives coincides with the number of stimulus intervals presented in the trial.
ISI	Inter-stimulus interval (in ms)
ITI	Inter-trial interval (in ms)
First factor	Value of the first factor set by the user.
First reversals	The number of reversals collected with the first factor.
Second factor	Value of the second factor set by the user
Second reversals	The number of reversals collected with the second factor
Reversal threshold	The number of reversals included in the calculation of the threshold.
Algorithm	Transformed up-down rule selected by the user
Block	Counter of the block number
Trials	Counter of the trial number. It starts from “1” at each new block.
Delta	The value that is varied in the threshold tracking. For example, in the frequency discrimination experiment it is the difference in Hz between standard and variable.
Variable	The value of the variable stimulus. For example, in the frequency discrimination experiment it is the frequency value of the standard plus the value of delta.
Variable position	A number representing the position of the variable in the trial
Pressed button	A number representing the response given by the participant
Correct?	A number that encodes whether the response was correct or incorrect
Reversals	A counter of the number of reversals collected thus far
Threshold (arithmetic mean)	The arithmetic threshold. This value is calculated for each block of trials.
Threshold (geometric mean)	The geometric threshold. This value is calculated for each block of trials.

## Footnotes

1 At the time of writing, the noise level of our office measures about 44 dBA.
